# Prediction of ablation zone disappearance after microwave ablation for the patients with papillary thyroid microcarcinoma using nomograph

**DOI:** 10.3389/fendo.2023.1145958

**Published:** 2023-08-02

**Authors:** Cai Hu, Shuang Liang, Huahui Liu, Jing Yang, Haiyu Kang, Sainan Guan, Ronghua Yan, Erjiao Xu

**Affiliations:** ^1^Department of Medical Ultrasonics, The Eighth Affiliated Hospital of Sun Yat-sen University, Shenzen, China; ^2^Department of Radiology, Peking University Shenzhen Hospital, Shenzhen, Guangdong, China; ^3^Department of Radiology, The Eighth Affiliated Hospital, Sun Yat-sen University, Shenzen, China

**Keywords:** papillary thyroid microcarcinoma (PTMC), microwave ablation (MWA), ablation zone, disappearance, nomograph

## Abstract

**Objectives:**

To construct a prognostic nomogram to predict the ablation zone disappearance for patients with papillary thyroid microcarcinoma (PTMC) after microwave ablation (MWA).

**Materials and methods:**

From April 2020 to April 2022, patients with PTMC who underwent MWA treatment were collected retrospectively. Ultrasound (US) or contrast-enhanced ultrasound (CEUS) was performed at 1 day, 1, 3, 6, 12, 18 and 24 months after MWA to observe the curative effect after ablation. The volume, volume reduction rate (VRR) and complete disappearance rate of the ablation zone at each time point were calculated. Univariate and multivariate logistic regression analysis were used to determine the prognostic factors associated with the disappearance of the ablation zone after MWA, and the nomogram was established and validated.

**Results:**

72 patients with PTMCs underwent MWA were enrolled into this study. After MWA, no tumor progression (residual, recurrence or lymph node metastasis) and major postoperative complications occurred. The ablation zone in 28 (38.89%) patients did not completely disappear after MWA in the follow-up period. Three variables, including age (odds ratio [OR]: 1.216), calcification type (OR: 12.283), initial maximum diameter (OR: 2.051) were found to be independent prognostic factors predicting ablation zone status after MWA by multivariate analysis. The above variables and outcomes were visualized by nomogram (C-index=0.847).

**Conclusions:**

MWA was a safe and effective treatment for PTMC. Older patients with macrocalcification and larger size PTMCs were more unlikely to obtain complete disappearance of ablation zones. Incomplete disappearance of ablation zone was not related to recurrence.

## Introduction

With the rapid development of ultrasonic imaging technology, the detection rate of thyroid nodular, including thyroid carcinoma has been increasing in recent years. Papillary thyroid carcinoma (PTC) is the most common pathological type of primary thyroid cancer, accounting for 80%-85% of all thyroid malignant tumors (1, 2). PTC with diameter ≤ 1.0cm is defined as papillary thyroid microcarcinoma (PTMC) (3). Thyroidectomy is the main treatment means for PTMC (4). Due to the good prognosis of PTMC and the trauma caused by thyroidectomy and its impact on the quality of life, more and more patients with PTMC refuse surgical treatment. The 2015 American Thyroid Association (ATA) guideline recommend active surveillance (AS) for PTMCs (5). However, AS can lead to an increased psychological burden of patients, resulting in a decreased quality of life (6). Ultrasound (US) -guided thermal ablation has been favored by many patients and clinicians for its advantages of less trauma, no scar, fast recovery and high effectiveness. It has been utilized in the treatment of a variety of malignant tumors, including liver, lung, kidney and also thyroid. Results of a systematic and meta-analysis involving 1289 patients, showed that the incidences of hoarseness, hypothyroidism, and postoperative pain were lower in the thermal ablation group, the hospitalization time was shorter, the postoperative effects were better than the conventional thyroidectomy group (7).

Microwave ablation (MWA) is an emerging technique that inactivates tumor cells primarily through protein denaturation caused by heat (8). Compared with other ablation techniques, MWA can cause larger ablated region, shorter treatment time, and more complete tumor ablation effect (9, 10). More and more guidelines have indicated that PTMC can be treated with ultrasound-guided thermal ablation (11). The recent studies have confirmed the safety and effectiveness of MWA in the treatment of PTMC (12–14).

According to the analysis of the clinical efficacy of 73 PTMCs treated with MWA, only 16 cases (21.92%) were completely absorbed. All patients achieved complete ablation, and no major complications occurred. However, local tumor progression occurred in 4 patients (5.48%) (12). According to relevant research results, local tumor progression may still occur after ablation in a few patients, even though the complete disappearance rate of tumor was as high as 84.5% (13). Conversely, local tumor progression may not occur even if the complete disappearance rate of tumor was as low as 16.70% (15). At present, there have been only a few studies predicting factors associated with the ablation zone disappearance after MWA for patients with PTMCs and evaluating the relationship between the ablation zone existence and the postoperative efficacy (such as tumor recurrence and lymph node metastasis).

Therefore, the aim of this study was to investigate the influence factors for the ablation zone disappearance of PTMC after MWA and establish a prognostic nomogram model to predict the probability of ablation zone disappearance. Meanwhile, the relationship between ablation efficacy and disappearance of ablation zone was evaluated during the follow-up period.

## Materials and methods

### Patients

From April 2020 to April 2022, patients with PTMC who underwent MWA were included in this study. The inclusion criteria were as follows: (1) All the patients were diagnosed as PTMC with diameter ≤10mm by fine needle aspiration (FNA). (2) There were no imaging evidences of capsule invasion, extrathyroidal extension, lymph node metastasis (LNM), or distant metastasis; (3) all patients refused surgical treatment or AS. The exclusion criteria were as follow: (1) incomplete clinical or image data; (2) history of thyroid surgery or neck radiotherapy; (3) follow-up period less than 6 months.

Each patient signed the informed consent form for ablation treatment. This retrospective study was approved by the ethics committee of the Eighth Affiliated Hospital of Sun Yat-sen University.

All patients were divided into completely disappearance group and incompletely disappearance group according to the remission status of ablation zone after MWA.

### Equipment and drugs

The MWA procedure were performed with a MWA system (MTI-5DT, Great Wall Medical Instruments, Nanjing, China) and a 19 G internal cooled MWA antenna with an active tip of 3 mm and 10 cm in length of needle handle. A power output of 15-30 W at 2450 MHz was routinely used.

MWA procedure were performed under US-guidance. A GE LOGIQ E9 (GE Healthcare, Pittsburgh, PA, USA) Ultrasound System with linear probes M6-15 (frequency of 6 to 15MHz) and L9 (frequency of 3 to 9MHz) was used for the guidance and evaluation during MWA procedure.

SonoVue (Bracco S.p.A Inc., Milan, Italy) was used as ultrasound contrast agent for contrast-enhanced ultrasound (CEUS) evaluation. CEUS was performed after bolus injection of SonoVue (1.5 ml), followed by 5 ml of normal saline flush. The perfusions of lesion and surrounding tissues were observed for at least 2 minutes.

### Pre-MWA assessment and data collection

Before MWA procedure, the comprehensive examination using US, CEUS, contrast enhanced computed tomography (CT) and laryngoscope were performed. The location, size, margin, shape, aspect ratio, echogenicity and composition and calcification were recorded by US examination. According to the location of the lesion, they were divided into bilateral thyroid (left lobe and right lobe) and isthmus. The maximum diameter was recorded as the tumor size. Calcification was divided into three categories: absent, microcalcification (≤1mm), and macrocalcification (>1mm) (16). The enhancement degree and pattern on CEUS were evaluated. The capsule invasion, extrathyroidal extension, lymph node metastasis, or distant metastasis were assessed and excluded by US and CT. FNA was performed on each nodule and the pathological result confirmed PTMC (Bethesda VI) before MWA. The thyroid function and thyroid protein (serum triiodothyronine, serum free thyroxine, and serum thyrotropin) were also tested and collected.

### MWA procedure

All the MWA procedures were performed by a senior doctor (XEJ) with more than 10-years’ experience of ablation under the guidance of US. Patients were placed in the supine position and their necks were extended. To accurately locate the tumor and complete coverage of the tumor, we generally performed a comprehensive scanning of the tumor, carefully observed the location of the lesion, and used surrounding anatomical landmarks (such as blood vessels, trachea, ipsilateral thyroid nodules, peripheral muscle layers, etc.) or surface projections to determine the position of the lesion. If necessary, we also measured the distance between the lesion and the surrounding capsules before ablation to further determine the position of the lesion, thereby accurately judging whether the ablation zone completely covered the tumor. Routine sterile skin preparation was performed with 1% lidocaine as subcutaneous local infiltration anesthesia guided by US. Following, a hydrodissection technique was used to protect major structures (carotid vessels, trachea, recurrent laryngeal nerve) from thermal injury. With the guidance of real-time US imaging, a microwave antenna was inserted into the thyroid to ablate the PTMC. Fixed ablation and “moving shot” ablation techniques were combined to cover lesion and expand ablation zone to obtain sufficient ablation margin (at least 3mm). For lesions < 5mm, we directly punctured the microwave antenna into the center of the lesion and use a fixed ablation method to obtain a sufficient ablation zone about 10-15mm in diameter. For lesions between 5mm and 10mm, we used “moving shot” ablation method from deep to shallow, from inside to outside, and from top to bottom to ensure complete ablation of the lesion. When the distance between the lesion and the thyroid capsule was ≥3mm, we would directly perform MWA treatment on the lesion, ensuring a ablation margin of at least 3mm. When the distance between the lesion and the thyroid capsule was less than 3mm, we would expand the ablation zone to the capsules. When the MWA procedure finished, CEUS was performed immediately after MWA to ensure the no-perfusion zone covered the lesion and its ablation margin sufficiently. Otherwise, immediate supplementary ablation was performed.

MWA parameters, such as ablation power, ablation time, and ablation energy were recorded. Throughout the procedure, we talked to patients intermittently to monitor the thermal injury of recurrent laryngeal nerve. After MWA, the patient’s neck was pressurized and cooled with ice bag for 2 hours, and the patient’s vital signs were closely monitored.

### Post-procedural evaluation and follow-up

Follow-up of laboratory examination and US examination were carried out at 1 day, 1, 3, 6, and 12 months after MWA and every 6 months thereafter. The size of the ablation zone was generally measured by conventional US to ensure the comparability of the measured values before and after. CEUS was employed to observe whether the lesion has been completely ablated one month after MWA to ensure complete necrosis of the lesion.

The ablation zones on US images were stored. The tumor volumes and Volume reduction rates (VRRs) were calculated and recorded. The volume reduction rate (VRR) was calculated by VRR(%)=([initial volume-final volume]×100%)/initial volume. The volume (V) of the tumor was calculated using the following formula: V=0.524abc, where a is the largest diameter, and b and c are the two perpendicular diameters. Complete disappearance was manifested as the ablation zone was invisible or “black line” sign (its volume could not be calculated) on US images (17). Complication and disease progression were recorded during follow-up. Suspected tumor progression (e.g., new lesions, lymph node metastases) was examined by CEUS or CT. Tumor progression was determined according to the following two criteria: (1) The new nodule in thyroid was confirmed to be PTC by FNA or CNB; (2) Lymph node metastasis proven by FNA, CNB, or thyroglobulin washout concentration (18).

### Statistical analysis

The statistical analysis was conducted with SPSS 24.0 software (IBM, Armonk, NY, USA) and R software (Version 4.2.0). Continuous data conforming to a normal distribution was presented as the mean ± standard deviation and range and was compared using Student’s t-test; nonconforming data was expressed as the median and range and was compared using the Mann–Whitney U test. Nonparametric Wilcoxon signed-rank test was used to compare the tumor volume and VRR at each follow-up time point. Categorical data were expressed as numbers (%) and were compared using a chi-square test or Fisher’s exact test. We performed a multivariate binary logistic regression analysis using the factors screened by univariate analysis to assess the probability of ablation zone disappearance and established a comprehensive prediction model. The ablation zone disappearance was set as 1, whereas the ablation zone non-disappearance was set as 0. Calcification type: 0, 1, and 2 represented macrocalcification, microcalcification, and no calcification, respectively.

The independent factors and outcomes predicted by the model were visualized by nomogram, and its performance and accuracy were evaluated. C-index and calibration curve were used to evaluate the calibration degree of nomogram. The receiver operating characteristic (ROC) curve was used to evaluate the discrimination performance of nomogram, and the corresponding area under the curve (AUC), sensitivity and specificity were obtained. *p*< 0.05 was considered statistically significant.

## Results

A total of 72 patients (20 males and 52 females) with 72 PTMCs were enrolled in this study from April 2020 to April 2022. The mean age was 35.51 ± 7.55 years (21-52 years). The mean initial maximum diameter was 6.14 ± 2.10mm (2.00-10.00mm). The median volume was 65.29 mm^3^(4.19, 471.60 mm^3^). The median ablation time was 120.00 seconds (60.00, 290.00 seconds). The median follow-up was 18.00 months (6.00, 24.00 months). Five lesions (6.94%) located in isthmus, 30 lesions (41.67%) located in the left lobe, and the other 37 lesions (51.39%) located in the right lobe. Among 72 PTMCs, there were 8 lesions (11.11%) presenting with macrocalcification and 22 lesions (30.56%) with microcalcification.

### Efficacy

**Figure 1:**
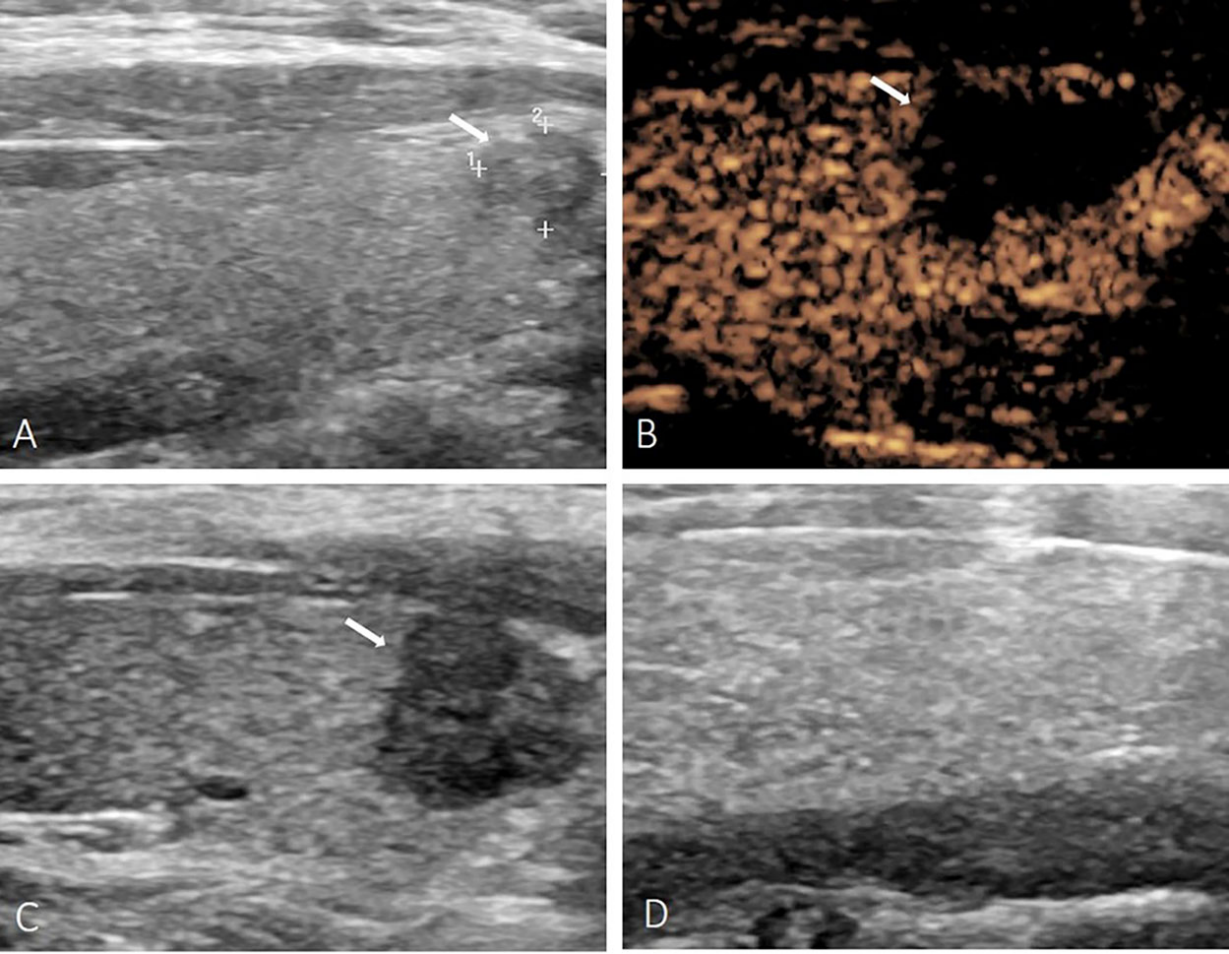
A 35-year-old female with PTMC. **(A)** Before MWA, US image showed a tumor (arrow) located in the right lobe. **(B)** At 1 day after MWA, contrast-enhanced ultrasound showed the PTMC was completely ablated. **(C)** At 1 month after MWA, the volume of the ablation area (arrow) was gradually reduced. **(D)** At 6 months after MWA, the ablation area was completely disappeared on US image.

The median pre-ablation volume of the lesions was: 65.29 mm^3^ (4.19, 471.60 mm^3^). Due to the expansion of the ablation margin, the median volume of the ablation zone was: 1166.93mm^3^(109.57, 4401.60mm^3^) at 1 day after MWA, which was significantly larger than the pre-ablation volume (*p*< 0.001). However, the volume significantly decreased gradually during the following 24 months after MWA. The results were shown in **Table 1**. Immediate CEUS after MWA revealed that the complete ablation rate of PTMC was 100% (72/72). During the follow-up period, complete disappearance of ablation zone occurred in 44 patients with a complete disappearance rate of 61.11% (44/72) (**Figure 1**). The complete disappearance rates of ablation zone at each follow-up time point were also shown in **Table 1**. Postoperative imaging examination and laboratory examination showed that no recurrence, residual, LNM and major complications occurred in patients with and without complete disappearance of ablation zones. Only 5 cases (6.94%) had minor complications after MWA, among which 1 case (1.39%) had cough and recovered within 10 minutes, and 4 cases (5.56%) had slight neck pain and resolved within 1 week. There was no ablation-induced hypothyroidism in each patient.

**Table 1:** Changes of volume, volume reduction rate (VRR) and complete disappearance rate at each follow-up timepoint.

Follow-up time (n)	Volume (mm^3^)	Volume reduction rate	Disappearance rate (%)	Cumulative disappearance rate (%)	*P* (VS volume before MWA
	Median (Range)	Median (Range)			
Before MWA (72)	65.29 (4.19, 471.60)	–	–	–	–
1 day (72)	1166.93 (109.57, 4401.60)	–	–	–	<0.001
1 month (72)	613.92 (22.01, 3428.72)	-710.22 (-6764.52, 44.42)	–	–	<0.001
3 months (72)	278.08 (5.97, 1982.46)	-187.34 (-3616.13, 95.66)	–	–	<0.001
6 months (72)	101.72 (0.00, 668.99)	9.63 (-2700.00, 100.00)	2.78	2.78	0.289
12 months (68)	32.73 (0.00, 398.08)	55.09 (-1268.89, 100.00)	23.61	26.39	<0.001
18 months (47)	13.20 (0.00, 225.92)	84.90 (-58.06, 100.00)	13.89	40.28	<0.001
24 months (29)	0.00 (0.00, 112.34)	100 (59.19, 100.00)	20.83	61.11	<0.001

N, the number of tumors. Range (minimum, maximum).

### Independent prognostic factors for complete disappearance of ablation zone

**Figure 2:**
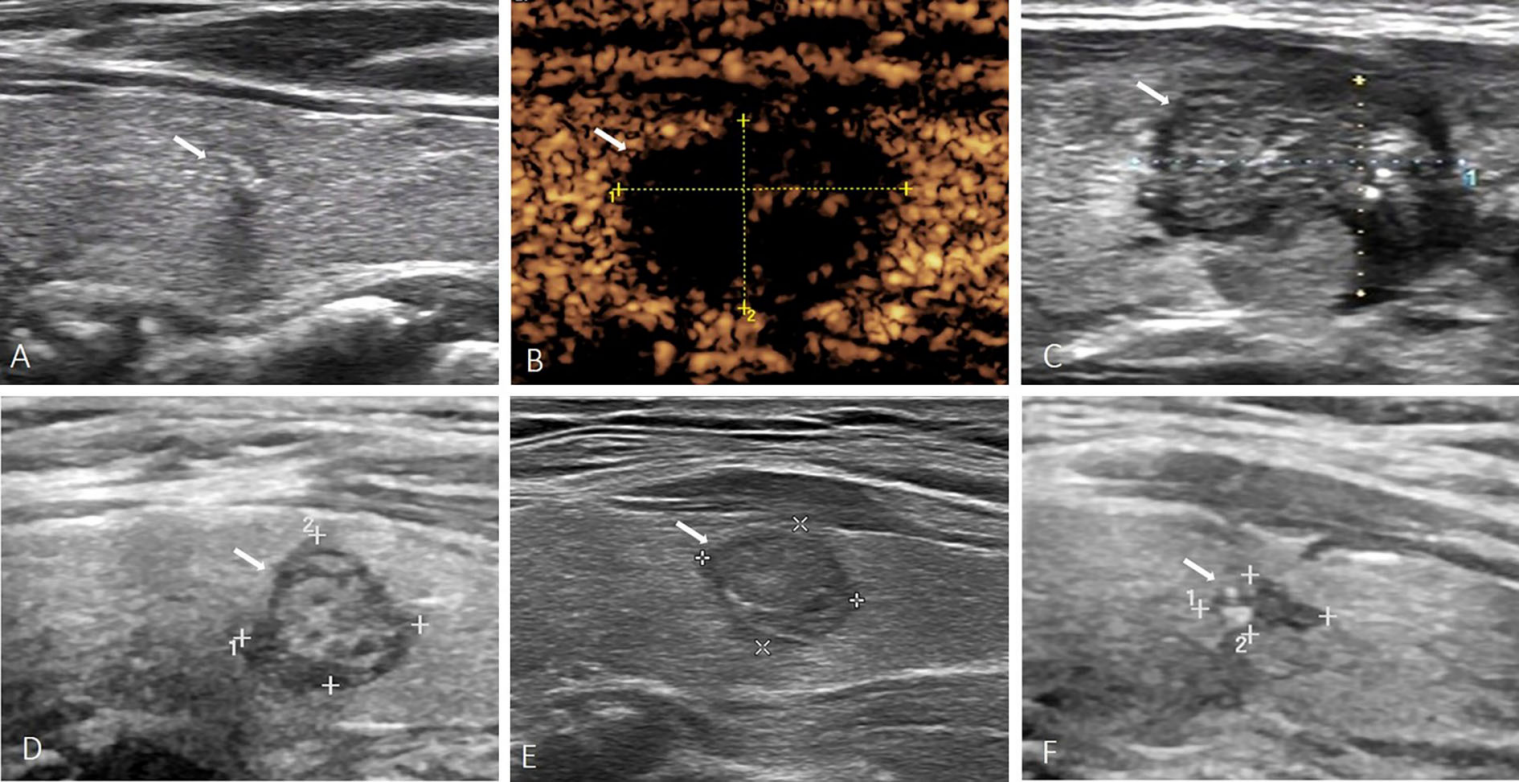
A 43-year-old female with PTMC. **(A)** Before MWA, US image showed a tumor (arrow) located in the left lobe with macrocalcification. **(B)** At 1 day after MWA, contrast-enhanced ultrasound showed the PTMC was completely ablated. **(C)** At 1 month after MWA, the volume of the ablation area (arrow) was not significantly reduced. **(D–F)** At 6, 12, 24 months after MWA, the volume of the ablation area (arrow) was gradually reduced, but the ablation area was not completely absorbed.

Univariate and multivariate Logistic regression analyses were performed to determine the independent factors for complete disappearance of ablation zone after MWA. The results of univariate analysis showed that age, initial maximum diameter, initial volume and calcification type, and volume of 1st day postoperatively were significantly correlated with the ablation zone status after MWA (**Table 2**). Multivariate Logistic regression analysis showed that age (odds ratio [OR], 1.216; 95% confidence interval [CI], 1.065-1.388; p=0.004), calcification type (OR, 12.283; 95% CI, 1.096-137.378; p=0.042), initial maximum diameter (OR, 2.051; 95% CI, 1.039-4.049; p=0.038) were independent prognostic factors predicting ablation zone status after MWA (**Table 3**). Patients with these three factors were more unlikely to obtain complete disappearance of ablation zones (**Figure 2**).

**Table 2:** Univariate analysis of clinical and ultrasound characteristics associated with regression status of ablation.

Characteristics	Disappeared N=44 (61.11%)	Un-disappeared N=28 (38.89%)	*P*
Sex, male/female	12/32	8/20	0.905
Age, years; mean ± SD	33.57 ± 5.81	38.57 ± 8.96	0.006
Maximum diameter, mm, mean ± SD	5.53 ± 1.98	7.09 ± 1.96	0.002
Volume, mm^3^#	48.10 (4.19, 471.60)	124.81 (11.90, 398.01)	0.002
Location, isthmus/left lobe/right lobe	3/17/24	2/13/13	0.862
Echogenicity, hypoechoic/isoechoic/hyperechogenic	32/8/4	20/5/3	0.975
Calcification type, no calcification/microcalcification/macrocalcification	29/14/1	13/8/7	0.012
Boundary, clear/unclear	26/18	17/11	0.891
CEUS, hypo-enhancement/iso-enhancement/hyper-enhancement	26/13/5	17/8/3	0.250
Volume at 1st day (mm^3^) #	922.88 (109.57,4401.60)	1407.46 (389.76, 4373.63)	0.014
Ablation time (s) #	118.00 (60.00, 290.00)	120.00 (80.00, 275.00)	0.395
Ablation power (W) #	25.00 (20.00, 30,00)	25.00 (20.00, 25.00)	0.225
Ablation energy (J) #	2950.00 (1300.00, 7250.00)	3000.00 (1800.00, 6000.00)	0.858
Follow-up (month) #	18 (6-24)	12 (6-24)	0.244

^#^Median (minimum, maximum).

**Table 3:** Multivariate analysis associated with complete disappearance of ablation zone.

Factors	OR	95%CI	*P*
Age	1.216	1.065 1.388	0.004
Maximum diameter	2.051	1.039, 4.049	0.038
Calcification type	12.283	1.096, 137.378	0.042

### Construction and validation of nomogram

**Figure 3:**
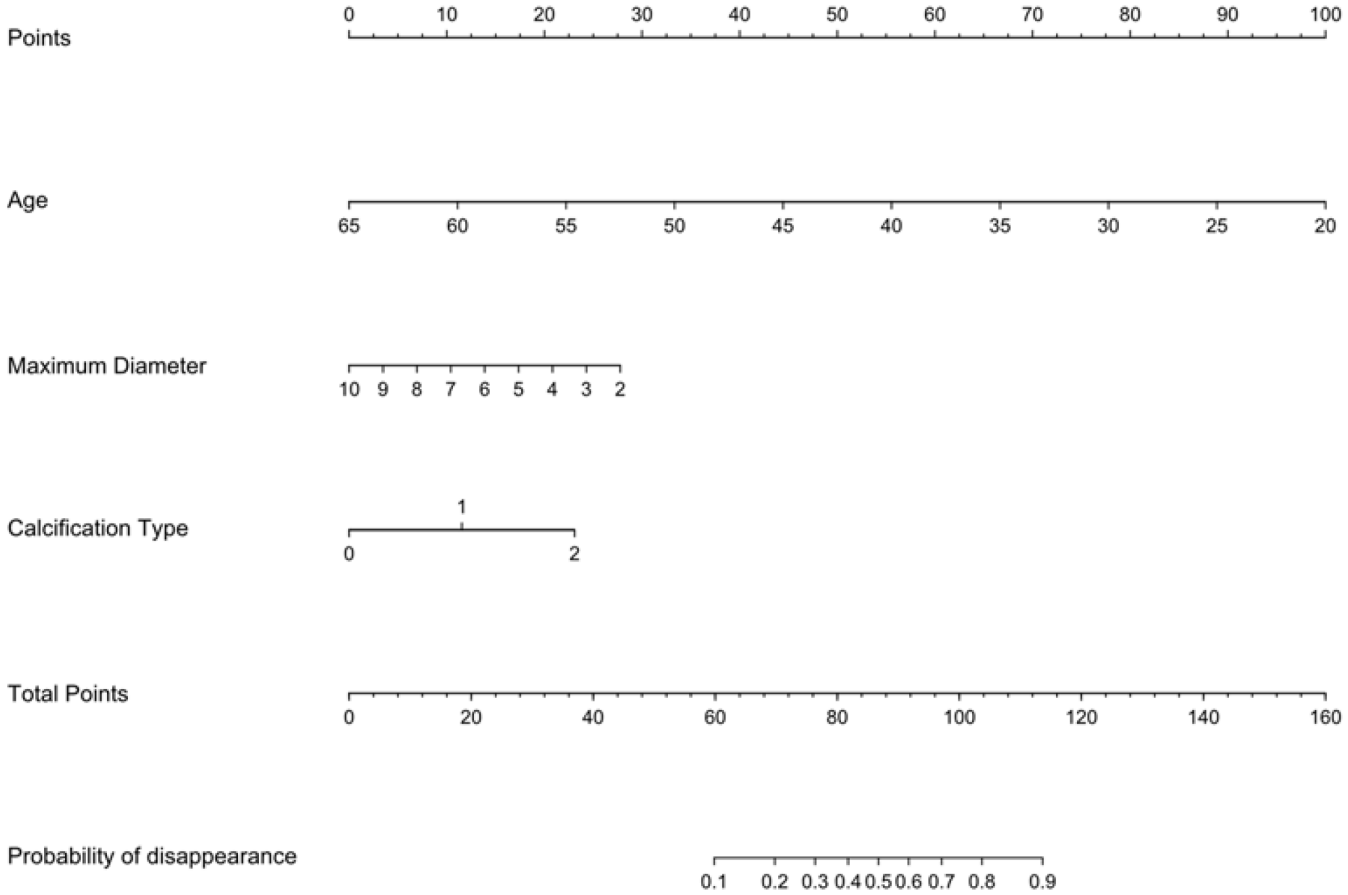
Nomogram for predicting the disappearance of the ablation zone for patients with PTMC after MWA.

**Figure 4:**
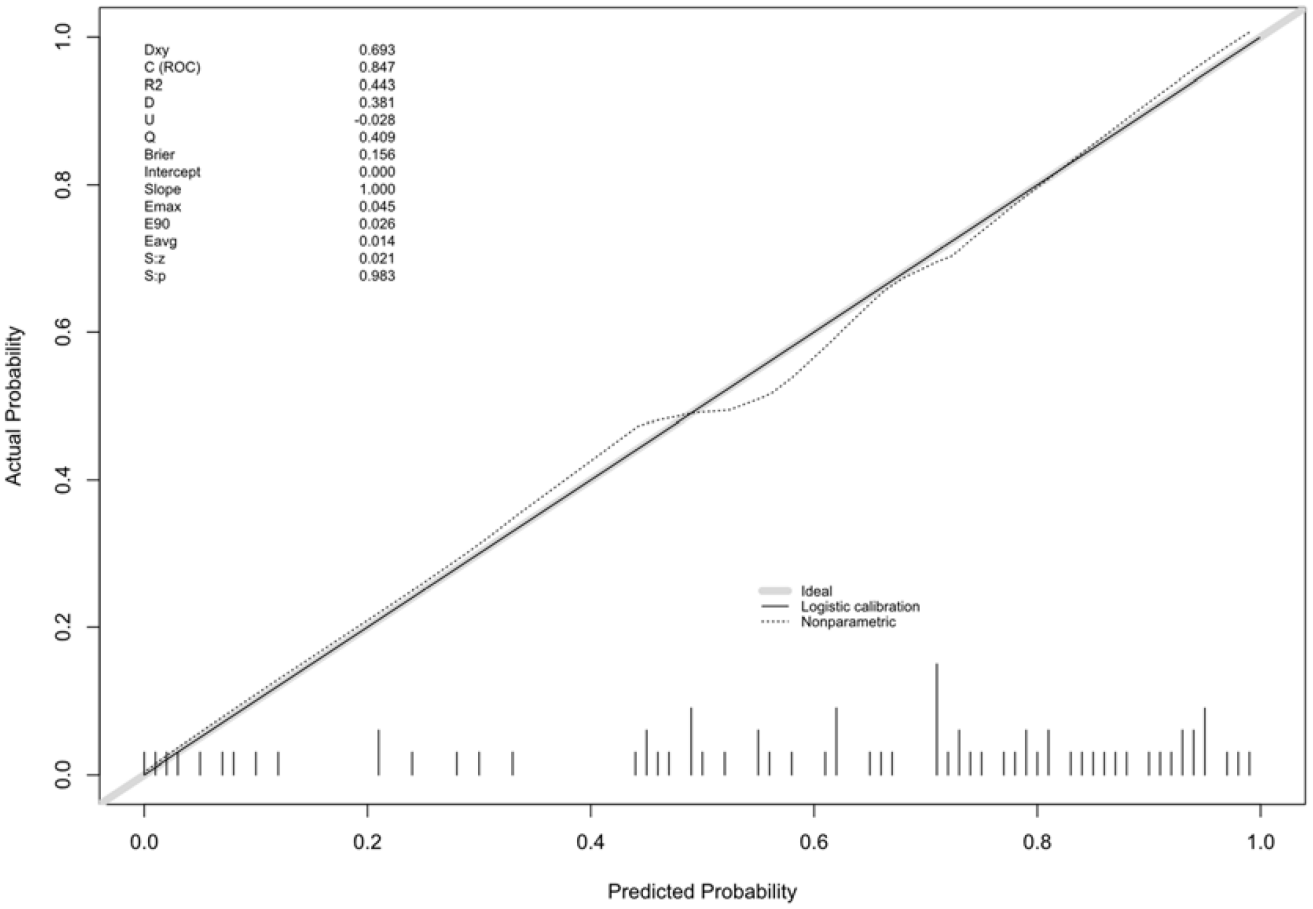
Calibration curve for predicting the ablation zone status for patients with PTMC after MWA.

**Figure 5:**
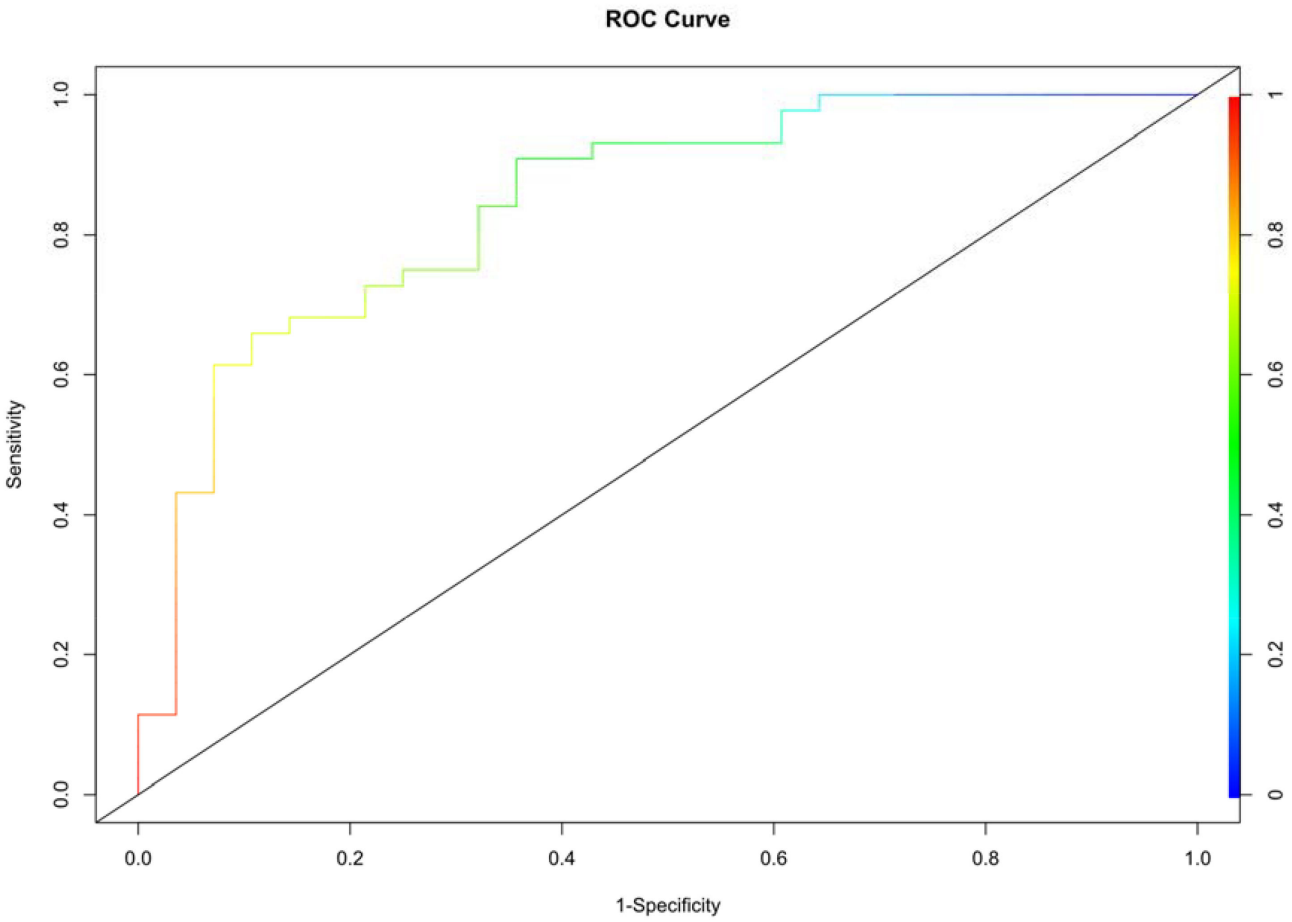
ROC curve for predicting incomplete disappearance of the ablation zone.

Multivariate logistic regression analysis was performed to determine the probability of ablation zone disappearance, and a nomogram was constructed (**Figure 3**). Each factor (age, calcification type, initial maximum diameter) can be converted into a corresponding score according to the nomogram. By calculating the total score of the three variables, the probability that the ablation zone disappeared completely for each patient after MWA could be provided. According to the calculation results of R software, the model has passed the calibration degree test (S:p =0.983> 0.05), and the C-index was 0.847. The above results indicated that the model had a good calibration degree (**Figure 4**). The AUC value of this model was 0.847, cutoff value was 0.499, sensitivity was 90.91%, indicating that this prediction model had satisfactory diagnostic efficiency and higher sensitivity (**Figure 5**).

## Discussion

This present study revealed that volume of ablation zone after MWA gradually decreased and VRR gradually increased during the follow-up period, the median VRR was 100% (59.19, 100%) at 24 months of follow-up, which was similar to previous studies (8, 19, 20). A systematic review and meta-analysis included a total of 12 eligible studies, with 1284 PTMCs. The study reported that volume of ablation zone decreased significantly over time after thermal ablation, with complete disappearance rates ranging from 57.3% to 76.2% (21). The complete disappearance rate of ablation zone in this study was 61.11% which was also within this range. Previous studies have shown that the tumor recurrence rate after MWA varied from 0.5% to 4.2% (12–14, 19). The rate of LNM varied from 0.1% to 2.0% after ablation (20–22). The incidence of total postoperative complications after RFA and MWA was 2.38% and 11.5%, respectively, and the incidence of major complications was 1.35% and 5.1%, respectively (23). Compared with RFA, MWA may cause more postoperative complications, but most of them were mild, including cough, transient hoarseness, burning sensation, toothache, anterior neck discomfort, and neck swelling (6). In our study, no lymph node metastasis, local recurrence, or major complications occurred during a median follow-up of 18 months. Only 5 cases (6.94%) had minor complications and recovered within a short period of time after MWA. These preliminary results in our study indicated that ultrasound-guided MWA was a feasible, safe and effective treatment for PTMC.

In a previous study, He et al. investigated the influence factors for the absorption of the ablation zone in the patients with PTMC after RFA. The results showed that the patient’s age, gender, calcification type and RFA ablation energy were independent risk factors. They suggested that older women with macrocalcification and higher energy exposure were not easy to obtain complete absorption of ablation zone within one year after RFA (24). In our study, we focused on the influence factors for the absorption of the ablation zone in the patients with PTMC after MWA. Our results were similar to the results of He et al (24)., but not identical. We suspected that this difference might relate to the utilization of thermal ablation techniques. Compared to RFA, MWA was characterized by higher intra-tumoral temperature, faster heating, larger ablation volume and short ablation time (25–29). The process after thermal ablation were aseptic inflammation, fibrosis of necrotic tissue and absorption of necrotic material (12). A relevant study (30) suggested that the decline in immune function with age might affect the immune system’s ability to respond to pathogens and repair damaged tissue, which might be the reason why ablation zone was difficult to absorb completely with age. In this study, we enlarged the ablation zone of the PTMC at least 3mm. Therefore, the larger the initial maximum diameter of lesion was, the larger the ablation zone would be, which was negatively correlated with complete absorption of the ablation zone. This result was consistent with the result of Lu et al (12). In addition, the lesions with macrocalcification were more difficult to be fully absorbed than those without calcification and those with microcalcification. The previous researches showed that microcalcification had no significant effect on the absorption of lesions, and the complete absorption rate reached 100%. While the complete absorption rate of lesions with macrocalcification was only 74.28%. Even if the follow-up period was as long as 36 months, lesions with macrocalcification were still difficult to be fully absorbed (13, 17). This might be related to the fact that the macrocalcification could not be ablated at high temperature.

In this study, we constructed a nomogram to predict the ablation zone status after MWA according to the multivariate logistic regression analysis of the three risk factors. The AUC value of this model was 0.847 which indicated satisfactory diagnostic efficiency to evaluate whether the ablation zone complete disappearance or not. In the cutoff value of 0.499, the sensitivity was 90.91%. This result suggested that the model has a better ability to judge the complete absorption of ablation zones. Meanwhile, whether the ablation zone disappeared completely or not is not directly related to the prognosis after MWA. The ablation zone did not disappear completely, which did not mean easy recurrence or metastasis after MWA.

There were several limitations in this study. This was a retrospective single-center study with small sample size. The follow-up period was not long enough and the nomogram model had not validated externally. A multi-center studies with larger sample size and longer follow-up period is necessary.

In conclusion, age, initial maximum diameter and type of calcification were related to the complete absorption of ablation zone for patients with PTMC after MWA. Whether the ablation zone disappears completely after MWA could be predicted by the nomogram we conducted. In addition, there is no correlation between recurrence or metastasis and whether the ablation zones disappear completely.

## Data availability statement

The raw data supporting the conclusions of this article will be made available by the authors, without undue reservation.

## Ethics statement

The studies involving human participants were reviewed and approved by the ethics committee of the Eighth Affiliated Hospital of Sun Yat-sen University. Written informed consent for this retrospective study was waived while written informed consent for microwave ablation procedure and contrast enhanced ultrasound examination was obtained from each patient.

## Author contributions

CH and SL interpreted the data and wrote the manuscript. CH, SL, HL, JY, HK and SG collected the patient data and performed the statistical analysis. RY and EX designed the study, and EX performed ablation procedure. CH and SL are co-first authors. RY and EX are co-corresponding authors. All authors contributed to the article and approved the submitted version.
